# The Anaerobic Fungi: Challenges and Opportunities for Industrial Lignocellulosic Biofuel Production

**DOI:** 10.3390/microorganisms9040694

**Published:** 2021-03-27

**Authors:** Luke M. G. Saye, Tejas A. Navaratna, James P. J. Chong, Michelle A. O’Malley, Michael K. Theodorou, Matthew Reilly

**Affiliations:** 1 Department of Biology, University of York, York YO10 5DD, UK; lmgs502@york.ac.uk (L.M.G.S.); james.chong@york.ac.uk (J.P.J.C.); 2 Department of Agriculture and the Environment, Harper Adams University, Newport TF10 8NB, UK; 3 Department of Chemical Engineering, University of California, Santa Barbara, CA 93106, USA; tejasn@ucsb.edu (T.A.N.); momalley@ucsb.edu (M.A.O.)

**Keywords:** anaerobic fungi, dark fermentation, lignocellulose, bioethanol, biohydrogen, biofuel, pretreatment, photofermentation, microbial electrolysis cell, methanogenesis

## Abstract

Lignocellulose is a promising feedstock for biofuel production as a renewable, carbohydrate-rich and globally abundant source of biomass. However, challenges faced include environmental and/or financial costs associated with typical lignocellulose pretreatments needed to overcome the natural recalcitrance of the material before conversion to biofuel. Anaerobic fungi are a group of underexplored microorganisms belonging to the early diverging phylum Neocallimastigomycota and are native to the intricately evolved digestive system of mammalian herbivores. Anaerobic fungi have promising potential for application in biofuel production processes due to the combination of their highly effective ability to hydrolyse lignocellulose and capability to convert this substrate to H_2_ and ethanol. Furthermore, they can produce volatile fatty acid precursors for subsequent biological conversion to H_2_ or CH_4_ by other microorganisms. The complex biological characteristics of their natural habitat are described, and these features are contextualised towards the development of suitable industrial systems for in vitro growth. Moreover, progress towards achieving that goal is reviewed in terms of process and genetic engineering. In addition, emerging opportunities are presented for the use of anaerobic fungi for lignocellulose pretreatment; dark fermentation; bioethanol production; and the potential for integration with methanogenesis, microbial electrolysis cells and photofermentation.

## 1. Introduction

New approaches are needed to reduce the use of fossil fuels and harness the global abundance of lignocellulosic biomass for biofuel production. Lignocellulosic feedstocks can be converted to biofuels but they require costly and environmentally damaging pre-treatments in order to overcome their inherent recalcitrance to degradation [1,2]. Anaerobic fungi, belonging to the phylum Neocallimastigomycota, may provide a green solution because they have an unprecedented ability to deconstruct crude lignocellulose [3,4] and are able to convert polymeric plant cell wall components to H_2_ [5] and ethanol [6]. This group of organisms can also produce volatile fatty acids (e.g., acetic and formic acid [5]) which are suitable substrates for additional downstream biofuel production.

Anaerobic fungi are commonly found in the digestive tracts of large mammalian herbivores, including many important livestock and companion animal species such as cattle, sheep, goats and horses. Prior to their correct affiliation [7,8,9] zoospores of anaerobic fungi were mistakenly classified as protozoa. *Callimastix frontalis* and *C. equi* zoospores, found in horse intestines, were both described as polyflagellated protozoans [10,11] and placed in the same genus as *C. cyclopsis*, a parasite of freshwater copepods [12,13]. Monoflagellated zoospores, found in ruminants, were also recognised as protozoa and assigned to the protozoan genera *Piromonas* and *Sphaeromonas* [10,14]. The discovery that *C. cyclopsis* was a fungal pathogen (belonging to the *Blastocladiomycota*) led to the transfer of *C. equi* and *C. frontalis* to a new protozoan genus, *Neocallimastix*, with *N. frontalis* as the type species [15]. Following the seminal work of Orpin [7,8,9] and their correct assignment as fungi, based upon the ultrastructure of their motile zoospores, anaerobic fungi were initially placed in the order Spizellomycetales but later transferred to their own order, the Neocallimastigales, in the phylum Chytridiomycota [16]. Genetic analysis identifies that Neocallimastigomycota is a distinct basal clade of the chytrids [17]. The order, which now houses 18 different genera of anaerobic fungi, was therefore raised to the level of a phylum, the Neocallimastigomycota, in 2007 [17,18].

The Neocallimastigomycota, Blastocladiomycota and Chytridiomycota are all closely related. Although fungi in the latter two phyla are aerobic and mostly found in fresh water and wet soils (some are parasitic), fungi in all three phyla display similarities in their adaptations to an aquatic lifestyle. These adaptations include a dependence on zoospore release in the aquatic environment for dispersal (via asexual reproduction) and similarities in vegetative thallus morphology, including the ability to grow on and within surfaces and substrates. Additionally, a dormant phase has been observed in all three phyla, where the fungi can survive relatively adverse conditions, sometimes for many months [19,20,21]. However, the Neocallimastigomycota differ from the blastoclades and chytrids in their anaerobic lifestyle and flagella apparatus. They also possess hydrogenosomes and a complete absence of mitochondria [19,22,23]. From an evolutionary perspective, these three phyla are basal fungal clades with species and genera that are the direct decedents of some of the earliest diverging fungal lineages. It has been proposed that that the Neocallimastigomycota diverged from other primitive aquatic fungi during the late Cretaceous period when grasses and grazing mammalian herbivores first appeared [24].

Due to their highly effective ability to convert lignocellulose into biofuels and biofuel precursors, anaerobic fungi are biotechnologically interesting. In this review, the challenges and opportunities associated with exploiting anaerobic fungi for the purpose of industrial biofuel production are discussed. Prior to discussing the challenges and opportunities for their exploitation in the biofuel industry, a review of the niche anaerobic fungi occupy in the mammalian digestive tract is presented, drawing in particular upon the substantial amount of literature involving ruminant livestock. This is necessary in the context of this review, as an in-depth appreciation of their natural niche will assist in developing appropriate methodologies for their exploitation in an industrial context.

## 2. The Gastrointestinal Tract of Herbivores

Ruminant nutrition and rumen function in domesticated livestock are mature and extensively researched scientific disciplines. This is because they are highly influential on productivity in farmed livestock and therefore impact farm profitability. Some of the more salient features of rumen function exemplify the precise nature of the ecological niche occupied by anaerobic fungi in the digestive tract ecosystem. They also provide a natural blueprint of what will be needed for in vitro exploitation of anaerobic fungi in an industrial context.

### 2.1. Rumen Function

There are two types of mammalian herbivores. Ruminant herbivores (cloven hoofed mammals), those in which a proportion of the gastrointestinal tract has been enlarged to produce a large fore-stomach (the rumen and reticulum) in which microbial digestion precedes gastric digestion, and hind-gut fermenting herbivores such as horses and elephants. A common constituent of the diets of these animals is lignocellulose found in monocotyledonous grasses and herbaceous woody plants. In order to deconstruct and utilise these recalcitrant materials, mammalian herbivores rely on a complex microbial consortium, resident in the gastrointestinal tract, to produce the variety of enzymes needed to degrade complex lignocellulosic substrates. Plant biomass is effectively converted by the consortium to microbial biomass and fermentation end-products, thereby providing nutrition and energy for the host animal. The evolved mechanism for selective retention of plant biomass in the rumen enables ruminant herbivores to achieve extensive degradation of plant fibre. One consequence of selective retention is that the accumulation of plant biomass in the reticulo-rumen restricts feed intake, which requires this organ to be relatively large (with a volume of 100–150 L in cattle and approximately 10 L in sheep [25]). Microbial digestion in hind-gut fermenting herbivores occurs mainly in the caecum and large intestines and follows gastric digestion. In comparison to ruminants, where feed particles can be retained in the rumen for 58–65 h (cattle), feed particles pass through the digestive system of hindgut-fermenting mammals relatively quickly (24–48 h, horse), and recalcitrant plant materials are not therefore digested as extensively [26,27,28].

**Figure 1:**
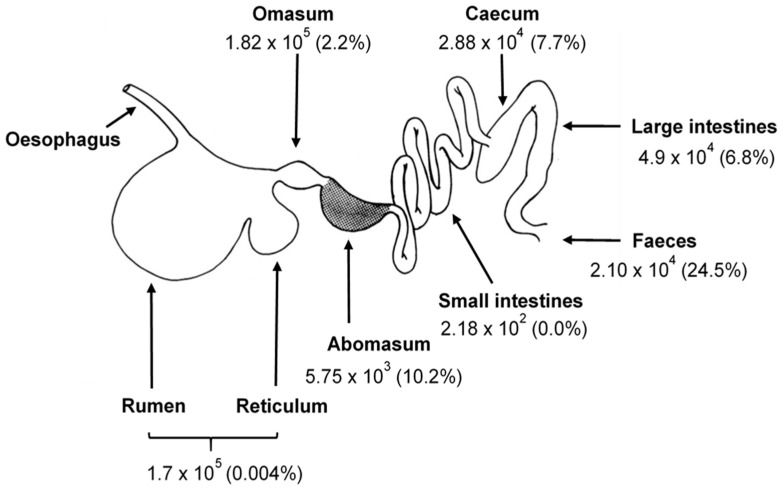
Diagrammatic representation of the ruminant digestive tract. Microbial digestion in the reticulo-rumen precedes gastric digestion which takes place in the abomasum, or true stomach. Values are presented for culturable anaerobic fungal populations (i.e., the number of thallus forming units per gram of dry matter, TFU g·DM^−1^) in digesta taken from each organ of the digestive tract of grass-silage fed, 8-month-old growing steers. Values in parentheses represent the percentage of the fungal population that survived and were culturable after 7 days of drying digesta or faecal contents at ambient temperature. Data taken from Davies et al. [20].

The ability to consume and digest plant polymers is most advanced in ruminant species. The foregut in these animals consists of four stomachs (Figure 1). The first three (the rumen, reticulum and omasum) are pre-gastric organs formed from modifications of the oesophagus. The fourth, the abomasum, is the site of gastric digestion, equivalent to the single stomach in monogastric mammals. The rumen contains an anoxic environment, homeostatically held at 39 °C and buffered at a pH of between 6 and 7. The digesta within are stratified into gas, liquid and solid; with solid particles of different sizes and densities. Feedstuffs do not just enter and exit the rumen. Instead, ingested feedstuffs travel along complicated flow-paths and are subjected to extensive mixing. Grazing ruminants typically swallow a feed bolus of plant material with minimal mastication. The rumen (or first stomach) receives the bolus and copious quantities of bicarbonate buffered saliva (6–16 L d^−1^ in sheep and 98–190 L d^−1^ in cattle [29]) from the oesophagus. The ingested biomass undergoes partial digestion in the rumen prior to being regurgitated, masticated (chewing the cud) and re-swallowed. Adequate mixing of digesting particles, microbial biomass, saliva and drinking water is assured by the grazing behaviour and synchronised rhythmic muscular contractions of the reticulo-rumen. Mastication, coupled with muscular contractions and the activity of microbial enzymes in the reticulo-rumen, ensures adequate digestion and particle-size reduction. Comminuted particles below a certain size (typically 1.5–2.0 mm in cattle [30,31]), liquids and free-floating microorganisms leave the rumen via the reticuluo-omasal orifice at the distal end of the omasum. The orifice, comprised of a series of “interlaced leaves” acts as a filtration and sieving mechanism, permitting the flow of liquids, free-floating microorganisms and smaller particles out of the rumen while preventing passage of larger, less digested particles. It is also the site where significant amounts of water and fermentation acids are absorbed by the animal. Thereafter, small digesta particles and microbial biomass undergo gastric digestion in the abomasum (true stomach) prior to passing to the hind-gut where further microbial activity can ensue. The retention times for liquids and small particles, including microorganisms, in the rumen are in the range from 10 to 24 h, whereas larger plant particles may remain in the rumen for 48–72 h, allowing more time for extensive microbial digestion of plant fibres [25]. Rumen function enables a large proportion of ingested plant biomass to be converted to microbial cells, gaseous and soluble fermentation end-products. Gaseous end-products (predominantly CO_2_ and CH_4_) are eructed via the oesophagus and soluble fermentation products enter the blood stream via absorption across the reticulo-rumen wall. Although ruminant and hind-gut fermenting herbivores have similar intestinal microbiomes [32], they digest plant tissues differently. Due to the different physiology of their gastrointestinal tracts, in hind-gut herbivory a greater portion of the nutrient supply to the animal is obtained from the contents rather than the walls of plant cells [33]. For a comprehensive account of the nutritional ecology of ruminants, please refer to Van Soest [34].

The brief description presented above serves to demonstrate the exquisite syntrophy that has evolved over millennia to enable ruminants to derive energy and nutrition from the consumption, microbial deconstruction and fermentation of structural plant polysaccharides. In essence, the rumen is an anaerobic fermenting environment that functions to enable retention of larger plant biomass particles while allowing passage of smaller particles, liquids and free-floating microbial cells to the lower gut. It is an open (continuous culture), high dry matter fermentation system in which plant biomass is preferentially retained based on particle size until masticated and digested to be small enough to pass out of the rumen. End-product toxicity in this high-substrate containing environment is mitigated by eructation of fermentation gases via the oesophagus and diffusion of aqueous fermentation end-products across the reticulo-rumen wall. An understanding of the intricacies of the herbivore digestion system is relevant because certain aspects of this natural blueprint will be required to accommodate industrial growth of anaerobic fungi for biofuel production.

### 2.2. The Relative Functional Role of Anaerobic Fungi

The plant fibre-degrading consortia in ruminants consists of anaerobic bacteria, archaea, protozoa and fungi. While each contributing species plays a part in the deconstruction of lignocellulose, due to the complexity of the community and ecosystem, it is challenging to assign and quantify relative roles to individual members of the consortium. It has been postulated that fibrolytic bacteria degrade particles of plant biomass by erosion, whereas rumen fungi degrade by invasion [35]. These two mechanisms of degradation may permit survival of both populations in a highly competitive ecosystem [36]. The concept of erosion versus invasion as a hypothesis for fungal survival is also the origin of the assertion that anaerobic fungi may contribute as primary colonisers of plant biomass [37]. This claim is supported by the observation that perennial ryegrass leaf blades in the rumen are colonised by anaerobic fungi within the first few minutes of their contact with rumen fluid [38]. The number of free-swimming fungal zoospores encountered in the rumen is typically within the range of 1 × 10^4^–1 × 10^5^ per mL of rumen fluid [7,8,9,39]. Given the temporal sequence of events in the fungal life cycle and the fact that many zoospores (10–120 per zoosporangium) are liberated from each vegetative thallus [40,41], it seems unlikely that the overall contribution made by anaerobic fungi to fibre deconstruction in the rumen will be large in comparison to the contribution made by fibrolytic bacteria. From the enumeration studies of Leedle et al. [42], it can be calculated that rumen bacteria typically outnumber fungal zoospores by up to 250,000:1. Acknowledging that many of these bacteria may not be fibrolytic, it might be expected that faster-growing and more numerous fibre-degrading species will survive at the expense of the less competitive anaerobic fungi. However, there is an increasing body of evidence to suggest that despite their lack of numerical abundance, anaerobic fungi make a contribution to the degradation of fibre in the rumen that is not negligible [36,43,44,45].

### 2.3. Life Cycle and Niche of Anaerobic Fungi

The anaerobic Neocallimastigomycota are aquatic, zoospore-producing fungi with a life history that has evolved over millennia to be uniquely adapted to a specific niche in the digestive tract ecosystem. Their life cycle involves a zoosporic dispersal phase, whereby freshly liberated zoospores are chemotactically attracted, settle and encyst on recently ingested plant biomass. Encysted zoospores germinate to produce a benthic, vegetative stage consisting of a rhizoidal or bulbous thallus which grows on the surface and throughout the substrate. Broadly speaking, anaerobic fungi can be subdivided between two distinct types based on the morphology of their fungal thallus. Monocentric fungi have a determinate life cycle, whereby the fungal thallus produces just one zoosporangium where all of the DNA is retained in the developing zoospores. When mature, the zoosporangium liberates its zoospores and the residual thallus, now devoid of nuclear material, autolyses without further development [40]. This finite growth habit is not unusual among zoosporic fungi. However, it is of consequence in an industrial context because it dictates that both zoospores and thalli are required for continuous biomass production. By contrast, nuclei are present in the bulbous or rhizoidal networks of polycentric fungi. Their life cycle is described as indeterminate because the thallus typically produces many zoosporangia per fungal thallus [46]. The development of a polycentric thallus with a nucleated rhizomycelium is considered as major step in fungal evolution, enabling the capacity for vegetative reproduction by fragmentation. In contrast to monocentric fungi, polycentric fungi have developed the ability to survive without the need for zoospore production. As both the vegetative and reproductive stages do not need to be accommodated, polycentric fungi may be more amenable to growth in industrial-scale bioreactors.

The life cycle in both aerobic and anaerobic zoosporic fungi is completed rapidly, resulting in the release of large numbers of zoospores. From studies conducted in the laboratory, it has been estimated that the duration of the anaerobic fungal life cycle is about 24–32 h [40,47]. However, under appropriate conditions in the animal, zoosporogenesis can take place as early as 8 h after encystment [48]. Research with ruminants suggests that the life cycle is synchronised to coincide with the feeding regime of the host [43,48,49]. Zoospores can respond chemotactically to diffusible components both in vivo in the animal in response to freshly ingested plant fragments [50], and in vitro, in the laboratory in response to soluble sugars and plant derived haemin and phenolic acids [51,52]. Evidence suggests that freshly ingested plant biomass triggers zoospore release from mature zoosporangia [48,50] and that within a matter of minutes, free–swimming zoospores rapidly colonise recently ingested biomass [38]. It is possible that zoosporogenesis is triggered by soluble constituents emanating from freshly ingested feed boli and that newly ingested feed boli are the primary sites of colonisation for fungal zoospores in the rumen. It is also possible that the life cycle of anaerobic fungi in the rumen may be restricted to the initial (primary) colonisation of freshly ingested feed boli and completed within a few hours, prior to mastication and re-swallowing of the regurgitated bolus. These points may be important considerations, relevant to bioreactor design because they suggest that the duration of the life cycle in vivo is quicker than that determined in vitro. They also suggest that the duration of the life cycle is not fixed but is influenced by environmental factors, particularly the type and temporal availability of plant biomass constituents.

A third stage in the fungal life cycle, an aero-tolerant survival stage, has been reported to occur when conditions for vegetative growth in the rumen become less favourable [20,21]. Research suggests that these survival structures are thick-walled, aerotolerant, desiccation-resistant zoosporangium formed by a proportion of the anaerobic fungi leaving the rumen. They have been quantified in cattle in all organs of the digestive tract and in faeces [20] (Figure 1). They are reported to germinate when conditions become favourable again, either in the hind-gut or after defaecation and re-introduction to a new host animal [20,21,53]. These results [20,21,53] and the fact that viable zoospores have never been observed in ruminant faeces (M.K.T. unpublished observations) suggest that stress-tolerant sporangial structures may be important for the transfer of viable fungi between herbivorous hosts.

While anaerobic fungi occupy a niche in the gastrointestinal tract where environmental conditions are relatively constant, by contrast, many aerobic zoosporic fungi occupy ecological niches that are subjected to changing environmental conditions. They have therefore developed various ecological strategies to enable them to survive transiently stressful conditions [54]. Given their common ancestry, it is reasonable to expect that the anaerobic fungi will have evolved mechanisms similar to their aerobic counterparts that enable them to persist in a dormant state outside of their mammalian host. While stress-tolerant, anaerobic fungal-resistant structures have been identified in digesta samples and in faeces, the ability to produce and germinate them in laboratory culture has not yet been achieved. This aspect of their life cycle requires further research as the ability to manipulate viable stress-tolerant structures in vitro could alleviate the need for repeated sub-culturing and therefore simplify inoculation and maintenance of anaerobic fungi in industrial fermentation processes.

## 3. Process Engineering and Genetic Engineering

A combination of process engineering and genetic (molecular) engineering approaches can aid the successful transposition of anaerobic fungi from their natural habitat in herbivores to effective exploitation in industrial biofuel production. The discipline of process engineering will be necessary to synthetically create a suitable habitat and environment in which an unnaturally large population of anaerobic fungi can resiliently prosper as a monoculture or co-culture in the absence of the host animal. The application of process engineering should include attention to design aspects such as the structure of fermentation vessels, solid and liquid amounts and retention times, suitable inoculation and plant biomass feeding regimes (batch or continuous). Genetic engineering provides the opportunity to manipulate anaerobic fungal cells and exploit their genetic potential for the purposes of higher product yields, increased environmental resilience and faster hydrolysis of lignocellulose material. Therefore, this section will discuss the current progress, challenges and future goals relating to achieving and optimising the industrial use of anaerobic fungi via both approaches.

### 3.1. Process Engineering

According to Dal Pont [55], process engineering can be summarised as the understanding and application of the fundamental principles and laws of nature that allow us to transform raw material and energy into products that are useful to society, at an industrial level. As yet, understanding of the anaerobic fungi falls far short of them being able to transform lignocellulosic substrates into biofuel energy products that are useful to society at an industrial scale. Moreover, it is important to underscore the significance of the anaerobic fungal niche in the mammalian digestive tract when considering opportunities and challenges for their biotechnological exploitation. On the one hand, an alternation of generations between reproductive motile zoospores and benthic, vegetative fungal thalli represent limitations (certainly for monocentric fungi) that must be accommodated if they are to be grown successfully in industrial processes. On the other hand, much can be learnt and potentially exploited (particularly from an engineering perspective) from a detailed understanding of the way in which the anaerobic fungi thrive and deconstruct lignocellulosic substrates in their natural habitat.

#### 3.1.1. Anaerobic Fungi in Anaerobic Digestion (AD)

Anaerobic fungi reside in and are easily isolated in culturable form from the digestive tract of large herbivorous mammals. In these environments, lignocellulosic substrates are abundant and oxygen is absent. Many other anaerobic environments contain an abundance of lignocellulose and might also support anaerobic fungi. For example, anoxic zones in landfill sites, anoxic muds and marshlands and purpose-built anaerobic digesters. Multiple studies have demonstrated that DNA extracted from these sites map unequivocally to the Neocallimastigomycota, suggesting that anaerobic fungi may be present and are not exclusively gut inhabitants [20,21,56,57,58,59,60,61]. However, the prevalence of large numbers of stress-tolerant survival structures in the faeces of mammalian herbivores means that their nucleic acid motifs will be abundant and widespread in nature. Therefore, it is to be expected that molecular signatures of anaerobic fungi will be found in a broad range of habitats outside of the gastrointestinal tract, wherever faeces are deposited. Detection of fragments of nucleic acid belonging to the anaerobic fungi in these locations should not be taken as evidence of their ability to undergo vegetative growth and reproduction. Where anaerobic fungi have been sought in landfill sites using culture methodologies, they have not been found [62].

In recent research, several studies have investigated the use of anaerobic fungi for bioaugmentation in industrial anaerobic digestion (AD) plants [57,58,63,64,65]. The rationale for inferring a role for anaerobic fungi in AD implies analogy with the digestive tract ecosystem. In both environments, complex molecules of plant origin are converted into simple organic molecules. The rationale is also cognisant of the fact that anaerobic fungi in their natural habitat form stable, syntrophic co-cultures with methanogenic archaea [5,66,67]. If anaerobic fungi could be successfully utilised in an AD plant, they could allow lignocellulose to become a major feedstock, representing an important step-change in the bioremediation process. Genetic motifs of anaerobic fungi have been found in industrial AD plants. In one study, 10 commercial plants in Germany were surveyed for transcriptional activity [57]. Anaerobic fungal 18S DNA was found, but only in plants that received cattle manure and of those, only two were found to contain GH5 endonuclease transcripts, suggesting metabolic activity. Others have also found genetic motifs of anaerobic fungi in manure-fed digesters, in landfill sites and in pond and stream muds adjacent to land grazed by livestock [58,59,60].

Anaerobic fungi are known to produce a survival stage that can exist for many months in dried livestock faeces [20,21,68]. They can also be readily isolated in culturable form from livestock manure and slurries [59,61]. Most isolates of anaerobic fungi studied in the laboratory have been obtained from livestock faeces. Thus, it seems inevitable that genetic motifs of anaerobic fungi will be detected in bioreactors, landfill sites or aqueous ecosystems where livestock manures are deliberately or accidentally introduced. It is therefore necessary to conduct this type of research in accordance with Koch’s postulates, to isolate, re-introduce and re-isolate viable cultures, before ascribing a role for anaerobic fungi in the AD environment, or indeed in any bioaugmentation study.

#### 3.1.2. Bioreactor Design and Habitat Engineering

Many of the techniques used to culture anaerobic fungi in the laboratory are based on methods developed by Hungate [69], Bryant [70], Hungate and Macey [71], and Miller and Wolin [72]. With relatively few exceptions these methods, together with the enumeration and growth determining procedures of Joblin [41] and Theodorou et al. [43,73], are used to routinely culture and maintain anaerobic fungi at bench-scale in the laboratory. This subject area was reviewed recently by Haitjema et al. [37]. In general, anaerobic fungi are grown at 39 °C without agitation in small batch cultures (of 10–100 mL culture volume) in thick-walled glass tubes or bottles sealed with gas-tight stoppers. In order to retain culture viability, anaerobic fungi must be maintained in sequential batch culture, with a transfer interval of between 2 and 7 days [37]. While some anaerobic fungi have been grown on defined media [74], better growth is obtained on complex media where sterile rumen fluid (10–15%) is an essential component of all such media. Problems associated with culture viability and the requirement for rumen fluid in culture media are noteworthy as barriers to growing anaerobic fungi in larger-scale bioreactors. The need for rumen fluid is a particular constraint to scale-up and research is required to elucidate those factors in rumen fluid that are necessary to stimulate fungal growth.

The first attempt to grow anaerobic fungi on a plant biomass concentration that was higher than that typically used in batch cultures was performed by Zhu et al. [75,76]. In their research, by continuously eluting growing cultures with fresh culture medium, they succeeded in growing an anaerobic fungus on increasing concentrations, up to 80 g dry matter (DM) L^−1^ of wheat straw. By using a multichannel peristaltic pump to deliver fresh culture medium to several culture bottles as spent medium was removed, these authors were able to monitor replicated cultures and make treatment comparisons. When compared with results obtained from conventional batch cultures, where the fungus is grown on just 10 g DM L^−1^ of wheat straw, their continuous-flow cultures produced up to 20 times more cell wall-degrading enzymes (CMCase and β-glucosidase) [75]. In comparisons involving anaerobic fungi grown on 80 g DM L^−1^ of wheat straw in batch or continuous-flow cultures, up to 30 times more cell wall-degrading enzymes were produced [76]. While just 5–9% of the wheat straw DM was lost in batch cultures grown on 80 g DM L^−1^, during the same incubation period, 52–56% was lost in comparable continuous-flow cultures [76]. The continuous-flow cultures described by Zhu et al. [75,76], although not representative of conventional continuous-culture systems where substrate as well as culture medium is removed, provided a simple and effective means of growing anaerobic fungi on high concentrations of plant biomass approximating those found in the rumen. The authors concluded that by using media flushing to remove the build-up of toxic fermentation end-products, the fungus was able to degrade considerably more wheat straw, produce significantly larger quantities of plant biomass degrading enzymes and survive for significantly longer periods of time in continuous-flow as opposed to batch cultures. In their 1997 publication, Zhu et al. [76] concluded that anaerobic fungi and continuous-flow cultures may have industrial potential. The effect of including in continuous-flow cultures, methanogenic and/or other non-methanogenic bacteria alongside anaerobic fungi offers intriguing possibilities and awaits further research. Important components of rumen fermentation, such as high DM concentrations, anaerobic conditions, selective retention of particulate matter, removal of toxic end-products and pulsed addition of substrate will need to be considered when developing suitable fermentation systems for the anaerobic fungi. 

#### 3.1.3. Solid Substrate Fermentation

**Figure 2:**
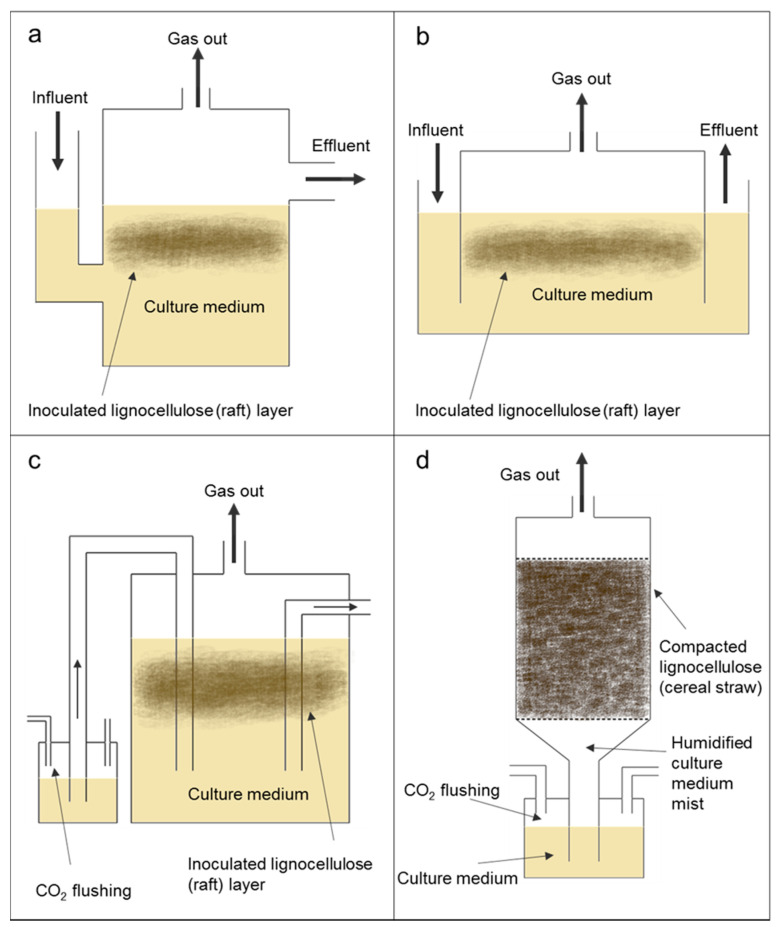
Schematic bioreactor and anaerobic digester designs for industrial-scale use of anaerobic fungi. A lignocellulose (raft) layer forms due to biomass floating as anaerobic fungi ferment their substrate: (**a**) up-flow anaerobic digester where anaerobic fungi are grown ± methanogens to produce CH_4_, H_2_ and CO_2_; (**b**) plug flow anaerobic digester; (**c**) continuous-flow bioreactor with intermittent substrate feeding; and (**d**) high dry matter (solid-state) bioreactor where anaerobic fungi ± methanogens grow directly on moist substrate. The bioreactor is flushed with CO_2_ humidified with culture medium. Substrate is batch fed and residual lignocellulose can be used downstream in biotechnological processes.

Solid substrate fermentation is a process in which microorganisms ferment a substrate in the absence of free water or with a very low free water content [77,78]. Unlike bacteria, filamentous aerobic fungi are able to grow on a substrate in the absence of free water by utilising the bound water in the substrate [79,80]. Aerobic fungi which grow on lignocellulosic substrates tend to grow in a linear rather than exponential manner [81]. Industrial applications for microorganisms such as *Trichoderma* and *Aspergillus* involve submerged culture bioreactors, but these fungi have been highly adapted and genetically modified for this purpose [82,83]. In their natural habitat, these fungi grow on solid substrates and are not submerged in culture media. Under these circumstances, and in this particular niche, the fungi require different enzymes, cellular structures and metabolites to those grown in submerged culture [84,85,86]. In recent years, there has been much interest in harnessing aerobic fungi for the purpose of solid substrate fermentation [78] and some of the adopted approaches may be applicable to the anaerobic fungi. Figure 2 presents, in schematic format, bioreactor designs that may be suitable for industrial-scale use of anaerobic fungi. While noting that the zoospores of anaerobic fungi exist in a liquid environment, their vegetative thalli grow directly on insoluble substrates and it may therefore be possible to adapt existing solid substrate fermentation methodologies to grow anaerobic fungi at industrial scale. The culture systems commonly used for solid substrate fermentation in industry are static bioreactors (fixed bed and perforated trays), agitated bioreactors (horizontal drum, continuously/intermittently pulsed) and mixing bioreactors (rotating drum) [78].

In comparison to submerged culture, solid substrate fermentations are less susceptible to bacterial contamination as most bacteria require a liquid environment in order to grow and/or form a biofilm on the surface of a substrate [78,87]. Hydrolytic enzymes in solid state fermentation systems are also less prone to substrate inhibition [78,87]. If secondary metabolites, enzymes or free sugars are the desired end-product in a solid-state fermentation, then the highly concentrated effluent produced serves to eliminate the need for costly additional downstream concentration steps [78,87]. By contrast, submerged fermentation has the advantages of easier control of parameters such as pH, temperature and separation of substrate from end products [88]. As many of the existing designs of solid-substrate fermenters are unsealed to the atmosphere, maintaining a strictly anaerobic environment will be a key challenge associated with adapting solid substrate fermentation for use with anaerobic fungi. Additionally, the absence of a liquid medium presents further challenges as the buffering capacity of the growth medium and the absence of reducing agents present the risk of oxygen toxicity killing the fungus. Nevertheless, it might be feasible to develop a continuous culture system based on a plug flow digester with a very high solids content, suspended in a highly concentrated growth media, similar to the bench-scale continuous-flow systems investigated by Zhu et al. [75,76].

### 3.2. Genetic Engineering

**Figure 3:**
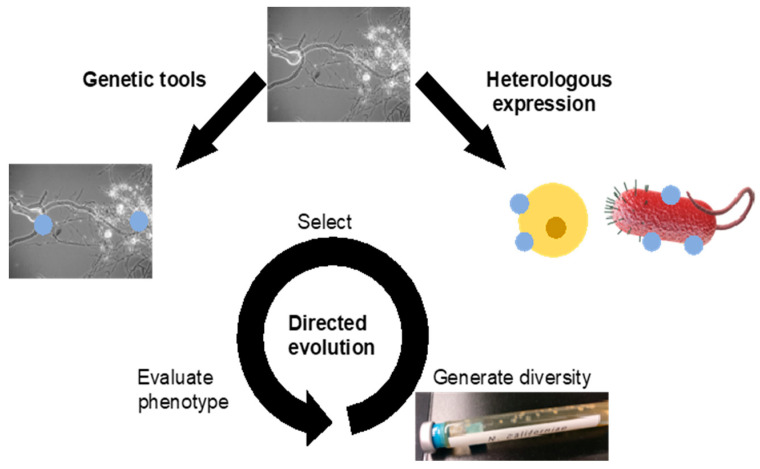
Anaerobic fungi show great potential for new genetic tool development and heterologous expression for biofuels production. Blue dots represent inserted or modified proteins, e.g., a fluorescent reporter attached to a knock-in cellulase. Fungi or heterologous hosts can be evolved for improved phenotypes, such as H_2_ production.

Anaerobic fungi have large genomes (~100–200 Mb) adapted for utilisation of plant matter and survival in the gastrointestinal tract of herbivorous mammals [37,89]. Solomon et al. [4] found that fungi obtained from horse, sheep and goat contained more genes encoding carbohydrate active enzymes (CAZymes) than any other microorganism. Many of these CAZymes are found in large multiprotein cellulosomes that allow the fungus to break down lignocellulosic biomass in a synergistic manner [37]. Despite historical and recent progress in this field, the composition of these extracellular enzyme–cellulosome complexes is not well described, and it is unclear whether fungal cellulosomes are predominantly secreted or bound to rhizoidal or bulbous structures [37,90,91]. With fungal genetic engineering to manipulate product selectivity and yields, anaerobic fungi show great potential for one-step processing of crude biomass. Realisation of this goal will require the development of robust genetic tools for anaerobic fungi (Figure 3). In parallel, the unique and diverse arsenal of enzymes used by these organisms [4] has spurred efforts to express native fungal genes in other hosts (heterologous expression).

As a framework for investigation, Wilken et al. developed a genome-scale metabolic model for *N. lanati*, an anaerobic gut fungus isolated from sheep faeces [74]. This model was validated by ^13^C metabolic flux analysis that identified the fluxes of carbon through glycolysis, tricarboxylic acid cycle, and in the hydrogenosome. For improved H_2_ production, future genetic engineering efforts may focus on directing flux through the hydrogenosome. This organelle and the pathways within it are not well characterised, with pyruvate ferredoxin oxidoreductase and/or pyruvate formate lyase potentially playing important roles in H_2_ production [74]. Future development of enzyme knockout strains can validate critical pathways and enable strain screening and evaluation to yield more productive enzyme and organism variants. Other potential biofuel targets include ethanol and butanol, produced by engineering strains with modified alcohol dehydrogenase and aldehyde dehydrogenase activities. Increased production of volatile fatty acids may also be beneficial when paired with other microorganisms to produce biofuels.

#### 3.2.1. Transformation

The obligately anaerobic nature and complex life cycle [92,93,94] are challenges towards genetic engineering of these fungi. There have been no reports of stable genetic transformation of anaerobic fungi so far. Transformation requires foreign DNA entry into the organism and either integration into host genomic DNA or maintenance through replicating structures such as plasmids or artificial chromosomes.

Given the expected low transformation efficiency of the techniques described here, it will be important to use a robust selection marker. The first report of transformation on an anaerobic fungus described transient expression of the β-glucuronidase gene under control of a putative enolase promoter using a biolistic device (gene gun) approach [95]. However, these experiments were conducted without any selection pressure for the delivered gene, and the blue pigment generated upon treatment with substrate post-transformation failed to appear 7 days after transformation. Anaerobic fungi have been reported to be sensitive to hygromycin B [96], and transformation with a resistance marker can potentially be a useful selection scheme. A scheme using the hph gene encoding a hygromycin B phosphotransferase is the most common selection method used in filamentous (aerobic) fungi [97]. Investigation into anaerobic fungal autotrophs can also be fruitful, as it would enable complementation strategies. The wild type strains of yeasts such as *Saccharomyces pombe*, *S. cerevisiae* and *Candida albicans* are sensitive to 5-Fluoroorotic Acid (5-FOA) due to native expression of orotidine-5-monophosphate decarboxylase (OMP decarboxylase, encoded by the URA3 gene). In yeasts, URA3 is involved in uracil biosynthesis, and URA3-deficient strains are dependent on uracil supplementation for growth. Anaerobic fungi can be grown in defined media without uracil supplementation [98] and published genomes contain a putative OMP decarboxylase [4,37,89], and are therefore likely to be sensitive to 5-FOA. A strategy involving knockout of the URA3 homologue and selection with 5-FOA merits further investigation for selection. 

Due to the fact that the vegetative thallus in monocentric anaerobic fungi is devoid of DNA and because fungal zoospores are reported to have relatively thin, non-chitinaceous, flexible cell walls [99], zoospores have been targeted as the most amenable life cycle stage for nucleic acid delivery and strain engineering. Calkins et al. [100] described a protocol to harvest zoospores from *Pecoramyces ruminantium* and later showed RNA interference-mediated knockdown of lactate dehydrogenase [101]. RNA interference (RNAi) has been observed naturally and is used in many organisms to decrease mRNA transcript number (and thus protein number) of targets [102]. Calkins and co-workers [101] identified genes required for RNAi in the genome of *P. ruminantium* and synthesised doubled-stranded RNA encoding a 21 base pair stretch in the lactate dehydrogenase transcript. They incubated this RNA with harvested zoospores and observed significant decreases in target gene expression (25% of untreated) and lactate production (14% of untreated) in propagated fungal mass. This work represents a promising proof-of-concept of metabolic engineering in anaerobic fungi and opens several interesting avenues of exploration. However, accompanying lactate dehydrogenase downregulation was an unwanted and non-specific downregulation of an additional 29 transcripts. Additional mechanistic investigation of siRNA targeting is needed, including understanding the duration of effect and generalisability to other genes and pathways.

A recent report by Swafford et al. [103] details the electroporation of the closely related blastoclades *Batrachochytrium dendrobatidis* and *B. salamandrivorans*. Electroporation is a widely used method of genetic transformation, in which target cells are exposed to a high electric field (typically 250–3000 V/cm) in the presence of DNA. The electric field is thought to cause temporary holes in the cell membrane and subsequent entry of DNA [103]. In the study by Swafford and colleagues, electroporation parameters (pulse shape, voltage and timing) were optimised for dextran entry and viability, resulting in 95% of zoospores taking up payload and a 41–71% survival rate, quantified by flow cytometry and motility, respectively. The authors observed that even without electroporation, some zoospores exhibited pericellular fluorescence due to dextran cell wall interactions, and analysis of electroporated cells showed intracellular signal, confirming uptake. Additionally, the authors note that the electroporation efficiency was highly dextran source-dependent, which has implications for extension to DNA transformation. Assembly of nucleic acids into polyplexes, as applied in the gene therapeutics field [104], may be necessary for high efficiency transformation of anaerobic fungi. Furthermore, uptake and persistence of DNA is dependent on long-term survival and division, and it is possible that electroporated zoospores may survive initially but fail to encyst and propagate. Careful quantification of zoospore propagation, through thallus forming unit (TFU) determinations [39], or gas pressure measurements [73], will be important for protocol validation especially when generating large and diverse gene libraries.

Other methods for nucleic acid delivery into non-model organisms are worth further exploration in their application to anaerobic fungi. *Agrobacterium tumefaciens* is a natural plant-targeting bacterium that has been used to integrate DNA into filamentous fungi, like *Aspergillus*. This system has been used to insert DNA into specific regions in the host DNA via CRISPR/Cas9. However, *Agrobacterium*-mediated transformation requires extended (>36 h) co-incubation at temperatures below 30 °C, whereas *Neocallimastix* grows best at 39 °C, and is capable of growth only between 33 °C and 41 °C [105]. The reconciliation of growth conditions is a necessary first step for developing a general *Agrobacterium*-mediated transformation protocol for anaerobic gut fungi.

#### 3.2.2. Heterologous Expression

While there is a growing effort to directly genetically manipulate anaerobic fungi, the challenges posed by these non-model organisms make the expression of genes of interest in model systems, like *Escherichia coli* and *S. cerevisiae*, appealing. A comprehensive list of reports of heterologous expression of anaerobic fungal proteins is available in Flad et al. [3]. Jones et al. [18] reported the expression and structural characterisation of anaerobic fungal glycoside hydrolases in *E. coli*, finding that arabinose-containing disaccharides were released by enzymatic digestion of plant-derived arabinan and arabinoxylan. In 2011, Jin reported the heterologous expression of endo-β-1,4-glucanase (EG) from an *Orpinomyces* strain in *T. reesei* [106]. Importantly, this required codon optimisation of the natively AT-rich anaerobic fungal gene. Wilken et al. describe a codon optimisation table, as well as amino acid and nucleotide-level abundance profiling of several fungal genomes, which would warrant consideration for construct design and engineering strategy development [107]. Seppälä et al. [108] expressed fluoride exporter proteins from several *Neocallimastix* strains in *S. cerevisiae* and found a higher activity variant than the wild type *S. cerevisiae* exporter, contributing to a higher fluoride tolerance.

Despite the rapid pace of progress in the heterologous expression of fungal enzymes, the complex, yet biotechnologically valuable anaerobic fungal cellulosome has yet to be expressed in a model organism, although en route to synthetic fungal cellulosome construction, dockerin-fused fungal enzymes have been expressed in yeast and *E. coli* [90]. Many heterologous proteins sourced from anaerobic fungi struggle to achieve soluble expression in model microbes, even after careful codon optimisation. This may be due to the inability of native-like post-translational modifications in the heterologous host, activation of stress responses in the host, or both [109]. Insertion of anaerobic fungal genes in currently more genetically tractable organisms can enable the use of typical protein engineering techniques such as directed evolution and structure-aided design. The creation of large protein libraries in *S. cerevisiae* and *E. coli*, in some cases exceeding 10^8^ variants, makes the high throughput screening of variants possible. Once an optimal variant is identified, it can be further refined and potentially retro-inserted into the original host, completing the development cycle. This methodology may be particularly relevant for anaerobic fungi, which are exceptional lignocellulosic degraders, but not highly genetically tractable, towards efficient production of biofuels.

## 4. Emerging Opportunities for Industrial Biofuel Production

The structure of lignocellulose and associated challenges relating to enzymatic access and hydrolytic degradation of its constituent polymers have been discussed frequently in the scientific literature (see, e.g., in [1,2]). Anaerobic fungi are considered to have good potential for use in a range of lignocellulosic biofuel production processes due to their natural vast array of CAZymes and repertoire of fermentation products which can be used either directly as fuel or as biofuel precursors [4,89]. The arsenal of lignocellulose degradation enzymes expressed by anaerobic fungi includes cellulases, β-glucosidases, hemicellulases, endoglucanases, exoglucanases and esterases [4,110]. However, it is not just the possession of an abundance of CAZymes that enable anaerobic fungi to efficiently degrade lignocellulosic substrates but also because their enzymes are selectively tethered to a large multi-protein complex, the cellulosome [4,37]. The ability to assemble, regulate and deploy CAZymes within a protein scaffold enables precision orientation of their catalytic domains towards the heterologous lignocellulosic substrates. Haitjema et al. [37] considers that the fungal cellulosome is an evolutionary chimeric structure that has evolved in anaerobic fungi by co-opting useful activities from bacterial neighbours within the gut microbiome.

This section introduces and discusses various scenarios in which anaerobic fungi could be utilised for lignocellulosic biofuel production. This includes consideration towards the use of anaerobic fungi in biological pretreatments and for consolidated bioprocessing purposes. Emphasis is placed on the assertion that anaerobic fungi perform dark fermentation and envisage their integration with emerging biofuel production systems.

### 4.1. Biological Pretreatment

Relatively few microorganisms can enzymatically deconstruct lignocellulosic substrates. Consequently, numerous microorganisms which have been identified as candidates for the production of biofuels, are unable to make use of the carbohydrates in lignocellulose without a pretreatment stage. Examples of such microorganisms include naturally occurring ethanologens, *S. cerevisiae* and *Zymomonas mobilis*, both of which lack cellulolytic activity [111].

Pretreatments are often energy-intensive or utilise potentially hazardous chemicals. At present, the use of lignocellulose material for biofuel production remains severely restricted due to the lack of effective, low-cost and environmentally friendly pretreatments [1,2]. In recent years, biological hydrolysis has begun to emerge as being potentially suitable for pretreatment purposes. Biological pretreatments can be divided into either the targeted use of hydrolytic enzymes extracted from culture broths or enzymatic hydrolysis during in situ microbial growth [112]. Most biological hydrolysis pretreatments to date have focused on the utilisation of aerobic fungi and their enzymes to degrade lignocellulose constituents [113,114,115,116]. According to Solomon et al. [4], the enzymatic repertoire of anaerobic fungi is superior to that of aerobic fungi, making them good candidates for use in biological pretreatment processes.

From the current literature, biofuels have yet to be produced at scale from lignocellulose hydrolysates created by pretreatments using enzyme cocktails derived from anaerobic fungi. However, Morrison et al. [117] reported the first use of an enzyme cocktail from an anaerobic fungus and successfully applied it to partially hydrolyse acid, alkali or ionic liquid treated corn stover and switch grass. A number of more recent studies have used in situ anaerobic fungal pretreatment of lignocellulose to improve biofuel production. Ranganathan et al. [118] tested the sequential growth of *P. ruminantium* and *E. coli* for the production of bioethanol from alkali pretreated corn stover. The anaerobic fungus was cultured on corn stover for 48 h before the addition of cycloheximide to inhibit growth. The authors reported that inhibition of the fungus resulted in the accumulation of free sugars from corn stover due to continued activity of residual enzymes derived from the fungus. After 14 days of saccharification, the biomass was inoculated with the ethanologenic *E. coli* strain KO11 and the authors reported a conversion efficiency of 14.1% of corn stover into bioethanol [118]. The investigators showed that the anaerobic fungal pretreatment was suitable for solubilisation of 9.91%, 17.19% and 10.6% of the fermentable sugars in switchgrass, sorghum forage and energy (sugar) cane, respectively [118]. Dollhofer et al. [63] performed a 7-day pretreatment of *N. frontalis* growth on hay before *Clostridia* dominated anaerobic digestion to produce biogas. The investigators found that the *N. frontalis* pretreatment significantly increased the rate of biogas production during anaerobic digestion [63]. Ferraro et al. [64] carried out a 72-h pretreatment on mushroom-spent wheat straw by using a microbial community that contained a pool of fermentative bacteria with and without two strains of anaerobic fungus. The authors concluded that the presence of *Neocallimastix* sp. and *Orpinomyces* sp. in the pretreatment improved the average yield of biomethane production from the mushroom spent wheat straw from 66.9 NmL-CH_4_ g-volatile-solid(VS)^−1^ to 117 NmL-CH_4_ g-VS^−1^.

The mechanism of enzymatic hydrolysis of lignocellulose by anaerobic fungi differs from that carried out by aerobic fungi. Aerobic fungi secrete free enzymes into their extracellular environment. In contrast, anaerobic fungi predominately synthesise cellulosome complexes which accommodate a multiplicity of interchangeable plant cell wall-degrading enzymes; this complex remains tethered to the cell membranes of their hyphae [119]. The physical attachment of cellulosome structures to rhizoid tips [120] may facilitate targeted localisation of CAZymes to their substrate. These attributes translate to potential benefits in hydrolysis times achieved by in situ growth of anaerobic fungi during the pre-treatment stage. Additionally, Hatakka [115] demonstrated that the supplementation of additional oxygen can reduce the pretreatment time required by aerobic fungi. The absence of any need for oxygen to facilitate growth of the anaerobic gut fungi (or the activity of their enzymes) has the potential to remove the costs and challenges associated with aeration of growth chambers.

A key disadvantage of in situ growth of fungi for pre-treatment purposes is the loss of feedstock carbohydrates to uptake, growth and cellular activity by the pretreatment microorganism, which causes a decrease in potential product yields from the receiving biofuel producing organism [121]. In addition, the accumulation of free sugars has been shown to repress the degradation of lignocellulose by anaerobic fungi [4,122,123]. If anaerobic fungi are to be used as an effective pretreatment, then a system to remove the hydrolysed sugars from the environment must be employed to limit their consumption by the fungus to what is essential for maintenance and to mitigate catabolite repression of their hydrolytic proteins. An alternative strategy to pretreatment is to develop the use of anaerobic fungi for consolidated bioprocessing and thus take advantage of their own capability to convert lignocellulose into bioethanol and biohydrogen fuels.

### 4.2. Consolidated Biofuel Production

Consolidated bioprocessing of lignocellulose into biofuels is the process of simultaneous saccharification of polysaccharides and the fermentation of solubilised sugars in a single bioreactor, ideally by a single microorganism. Consolidated bioprocessing is potentially advantageous over the use of a separated pretreatment stage for a number of reasons. First, the continual fermentation of newly solubilised sugars avoids their accumulation in the environment and the associated risk of product inhibition of CAZymes [91]. Second, the additional financial costs associated with equipment and operation of multiple bioreactors can be saved. Third, if a single species can be utilised and optimised for bioprocessing, then this provides a potential opportunity to achieve a higher ratio of biofuel to microbial biomass and thus greater specific product yields.

#### 4.2.1. Bioethanol Production

The potential use of anaerobic fungi for the consolidated production of bioethanol is worth exploring due to the combination of their CAZyme and cellulosome resources, an ability to utilise both 5C and 6C carbohydrates and their possession of ethanologenic metabolism [124]. It has been shown that a range of lignocellulose-containing substrates (e.g., wheat straw [124], bagasse [124], barley straw [6], energy cane [118], sorghum forage [118] and corn stover [118]) can be converted to bioethanol by anaerobic fungi. Examples of bioethanol production by anaerobic fungi are presented in Table 1, which has been compiled to give a limited overview of the variable and typically low concentrations produced that have been reported to date. Historically, the production of ethanol by anaerobic fungi has been reported when the metabolic flow of carbon has not been considered during fermentation studies designed to optimise the digestibility of forage, and for which the main objective was not the production of bioethanol. This lack of optimisation could be a contributing factor, explaining why bioethanol yields from anaerobic fungi are low in comparison to those from filamentous fungi such as *Fusarium oxysporum* [111] or *T. reesei* [125]. A key advantage for anaerobic fungi (over *F. oxysporum* or *T. reesei*) is not just their CAZyme repertoire but also their ability to carry out both lignocellulose degradation and bioethanol production in the absence of oxygen. This latter characteristic removes the need for intermittent switches between aerobic and anaerobic conditions within an industrial-scale bioreactor, which has previously been identified as problematic for maintaining upkeep of obligately aerobic fungi [125].

Although anaerobic fungi represent an interesting opportunity for the industrial consolidated production of bioethanol from lignocellulose, a number of challenges must be resolved to sufficiently improve the process. One prospective challenge is the product inhibition of anaerobic fungal growth that can be caused by the accumulation of ethanol [126]. The kinetic product inhibition constant for ethanol in relation to the growth of *N. frontalis* on cellulose was reported to be 222 mM (or 10.2 mg L^−1^) [126]. For comparison, half of the maximum ethanol product inhibition for *S. cerevisiae* has been shown to occur in ethanol concentrations of between 10 and 20 g L^−1^. Current challenges faced by consolidated bioprocessing of ethanol by anaerobic fungi include low and variable ethanol yields (Table 1) and the preferable formation of alternative fermentation end-products (e.g., formic acid, acetic acid and lactic acid) [126]. Potential avenues for overcoming these obstacles include upregulation, heterologous expression, induced mutations or selective pressure of genes relating to ethanol production or tolerance. There has been success in the use of some of these techniques for improving ethanol yields in aerobic fungi. For example, Stevenson and Weimer [127] isolated a strain of *Trichoderma* from cow dung and improved bioethanol production from cellulose from approximately 0.4 g ethanol L^−1^ to 2 g ethanol L^−1^. The authors achieved this increase after inducing mutations, facilitating parasexual fusion and increasing the availability of O_2_, although the actual cause for the increase was undetermined [127]. In another study, overexpression of the carbohydrate membrane transporter *Hx*t in *F. oxysporum* caused a 39% increase in consolidated bioethanol production from a mixture of wheat straw and bran [128]. By comparison, the development of anaerobic fungi for the purpose of consolidated bioethanol production remains a relatively understudied topic.

**Table 1:** Ethanol production by anaerobic fungi.

Fungal Isolate	Substrate	Ethanol Yield [µmol g^−L^] *	Reference
*Piromyces* sp., isolate E2	Cellobiose	50	[124]
*Piromyces* sp., isolate E2	Glucose	80	[124]
*Piromyces* sp., isolate E2	Fructose	80	[124]
*Piromyces* sp., isolate E2	Mannose	80	[124]
*Piromyces* sp., isolate E2	Lactose	14.77	[124]
*Piromyces* sp., isolate F1	Xylose	1920	[129]
*Piromyces* sp., isolate E2	Xylose	113	[124]
*Piromyces* sp., isolate E2	Xylan	84	[124]
*N. frontalis*	Cellulose	2310	[5]
*N. frontalis*	Cellulose	3750	[130]
*Piromyces* sp., isolate E2	Cellulose	157	[124]
*Piromyces* sp., isolate E2	Wheat straw	695	[124]
*Piromyces* sp., isolate E2	Wheat bran	891	[124]
*Piromyces* sp., isolate E2	Starch	157	[124]
*Pecoramyces ruminantium*	Switch grass **	540	[118]
*P. ruminantium*	Energy cane **	510	[118]
*P. ruminantium*	Sorghum **	560	[118]
*P. ruminantium*	Mixed prairie **	490	[118]
*P. ruminantium*	Corn stover **	1030	[118]

* Values quoted are with zero decimal places. ** Substrates were pretreated with 3% NaOH. All substrates were autoclaved prior to fermentation and fermented in batch culture.

#### 4.2.2. Dark Fermentation

To date, the potential uses of anaerobic fungi in industrial biogas production processes has been researched and discussed in the context of converting lignocellulose into precursors for biomethane production. Combining the growth of anaerobic fungi with methanogenic organisms to ultimately produce biomethane has been reviewed in detail by Dollhofer et al. [120]. However, the use of axenic cultures of anaerobic fungi to produce biohydrogen as an alternative to the symbiotic production of biomethane from lignocellulose has not been considered. The use of biohydrogen could be advantageous over biomethane in terms of fuel efficiency. This is because hydrogen has a higher heating value (HHV) of 141.9 kJ g^−1^ [131]. In comparison the HHV of methane is 61% lower at 55.5 kJ g^−1^ [131]. At present, the global production and consumption of H_2_ is approximately 70 Mt year^−1^ [132]. The gas is almost exclusively derived from reforming of fossil fuels and production causes 830 Mt of net CO_2_ emissions, annually [132]. Therefore, one of the barriers to a sustainable global H_2_ economy is the requirement for a low-cost, green method of production such as biological generation from lignocellulosic biomass. The use of anaerobic fungi for this purpose, with their hydrogen generating hydrogenosomes and unrivalled ability to deconstruct lignocellulosic substrates, merits detailed scientific exploration. Previously, the anaerobic production of H_2_ by bacteria or algae in the absence of light has been collectively referred to as “dark fermentation” [133,134]. Herein, and for the first time, the term dark fermentation is used while discussing the potential for industrial fermentative biohydrogen production by anaerobic fungi.

The production of H_2_ by axenic cultures of anaerobic fungi was first reported by Bauchop and Mountfort [5]. In that study, an anaerobic fungus from the ovine rumen was grown on cellulose in the presence and absence of methanogenic archaea. In the absence of methanogens, the authors reported H_2_ yields of 0.353 mol-H_2_ mol-hexose^−1^. Since the initial observations of Bauchop and Mountfort [5], the production of H_2_ has been detected during the growth of a range of anaerobic fungal species (Table 2).

Anaerobic fungi produce H_2_ in membrane-bound organelles known as hydrogenosomes [135]. Hydrogenosomes produce H_2_ gas by using protons as electron acceptors during mixed-acid fermentation of monomeric sugars (predominantly glucose and xylose) derived from cellulose and hemicellulose, to generate ATP. The reduction of protons is catalysed in the hydrogenosome by hydrogenase enzymes which are known to be highly sensitive to product inhibition by H_2_ [135,136]. In their natural habitat in the mammalian digestive tract, the accumulation of H_2_ is limited due to consumption and conversion by methanogens [137]. Therefore, methanogenesis is likely to facilitate the activity of hydrogenase and generation of H_2_ and ATP by anaerobic fungi [135].

Currently, the relationship between the partial pressure of H_2_ in the environment and metabolic shifts by anaerobic fungi, away from hydrogenosome fermentation pathways, has not been fully elucidated [129,138]. It is known that in the absence of H_2_-consuming organisms, anaerobic fungi increase production of alternative electron sinks to H_2_ (e.g., lactate, ethanol and succinate) [66,139,140] and suppress lignocellulose deconstruction [5,6,66,138,139,141,142,143,144,145]. Previous observations of reduced lignocellulose hydrolysis are supported by recent transcriptomic analysis which revealed that *Anaeromyces robustus* downregulated overall CAZyme production when grown axenically in comparison to when co-cultured with *Methanobacterium bryantii* [144]. Furthermore, H_2_ has previously been observed to inhibit the growth of a range of other fermentative microorganisms [136,146,147,148]. Therefore, it is hypothesised that the accumulation of H_2_ limits dark fermentation H_2_ yields from anaerobic fungi and that their industrial growth in the absence of H_2_-consuming species will require the integration of suitable technical solutions for in situ H_2_ removal.

**Table 2:** Anaerobic fungal H_2_ yields from dark fermentation.

Fungal Isolate	Substrate	H_2_ Yield [μmoL g^−1^] *	Reference
*Piromyces* sp., isolate E2	Cellobiose	54	[124]
*Neocallimastix* sp., isolate R1 **	Glucose	3464	[105]
*Piromyces* sp., isolate F1	Glucose	≈377 ***	[138]
*Piromyces* sp., isolate E2	Glucose	70	[124]
*Piromyces* sp., isolate E2	Fructose	161	[124]
*Piromyces* sp., isolate E2	Lactose	106	[124]
*Piromyces* sp., isolate E2	Mannose	88	[124]
*Piromyces* sp., isolate E2	Xylose	106	[124]
*Neocallimastix* sp., isolate R1 **	Xylose	8020	[105]
*N. frontalis*	Cellulose	2177	[5]
*Sphaeromonas communis*	Cellulose	2880	[143]
*Neocallimastix* sp.,isolate N1	Cellulose	2520	[66]
*Neocallimastix* sp., isolate N2	Cellulose	2600	[66]
*Piromyces* sp., isolate E2	Cellulose	2220	[66]
*Piromyces* sp., isolate R1	Cellulose	2460	[66]
*Piromyces* sp., isolate E2	Cellulose	159	[124]
*N. frontalis*	Xylan	≈2381 ***	[141]
*Piromyces* sp., isolate E2	Wheat Straw	2261	[124]
*Piromyces* sp., isolate E2	Wheat bran	1370	[124]
*Piromyces* sp., isolate E2	Bagasse	1957	[124]
*N. frontalis.*	Poplar wood chips	1984 ***	[149]
*Piromyces* sp., isolate E2	Xylan	134	[124]

* Values quoted are with zero decimal places. ** *Neocallimastix* sp., isolate R1 was classified *N. hurleyensis* [150] and subsequently reclassified as *N. frontalis* [151]. *** Calculated on assumption that H_2_ mL reported in referenced paper was stated at 1 atm. All substrates were autoclaved prior to fermentation and fermented in batch culture.

There are a number of technologies which have been applied at lab-scale to dark fermentation by bacteria to decrease product inhibition by H_2_ and significantly increase H_2_ production. Research in this area is ongoing but examples of these technologies include particular mixing regimes [152], gas sparging [153], ultrasonication [154], gas separation by membranes [155], the maintenance of low-pressure fermentation environments [156] and electrochemical removal [157]. For example, Mizuno et al. [153] demonstrated that the H_2_ yield from anaerobic communities growing on glucose could be increased from 0.85 mol-H_2_ mol-hexose^−1^ to 1.43 mol-H_2_ mol-hexose^−1^ by using a flow of N_2_ gas to continuously sparge the fermentation system. Other investigators have reported comparable improvements to H_2_ yields when sparging with CO_2_ [158]. In another study by Niño-Navarro et al. [152], it was reported that a selection between the use of two common impeller designs resulted in a greater than 2-fold increase in fermentative H_2_ productivity which the authors attributed to mass transfer of H_2_ gas from the liquid phase. More recently, Ramírez-Morales et al. [155] successfully implemented the use of polymeric membranes to separate H_2_ from the headspace gas created during fermentation by a microbial consortium native to tobacco wastewater. Operation of their novel membrane bioreactor caused a 16% rise in H_2_ production [155]. The development and application of H_2_ removal technologies to the growth of anaerobic fungi (in the absence of methanogenic archaea) is an area of research which requires scientific attention to assure that the maximum potential of these organisms for H_2_ production can be fully realised.

The yield of H_2_ from dark fermentation by other microorganisms is known to be significantly influenced by many other parameters including feedstock type, feedstock concentration, pH, temperature and species. Thus far, the reported production values for H_2_ from dark fermentation by anaerobic fungi remain low (Table 2). For example, the highest anaerobic fungal H_2_ yield from glucose of 3464 μmol g^−1^ (0.624 mol-H_2_ mol-hexose^−1^) [105] is towards the lower end of the typical range of between 0.57 and 2.80 mol-H_2_ mol-hexose^−1^ reported for bacterial dark fermentation [159]. Notably, anaerobic fungal H_2_ yields of 2261 μmol g^−1^ of wheat straw (54.3 NmL-H_2_ g^−1^) is comparable to a previous bacterial study that achieved a yield 58.78 NmL-H_2_ g^−1^ of alkaline pretreated wheat straw that had been supplemented with enzymes [160]. The current lack of process optimisation presents significant scope for H_2_ yield improvement from anaerobic fungi. Furthermore, a number of avenues exist to add further value to dark fermentation by making use of the soluble fermentation end-products via their biological conversion into additional H_2_ or CH_4_ biofuels.

### 4.3. Biofuel Production from Dark Fermentation Products

In addition to H_2_ and CO_2_ gases, anaerobic fungi have the capability to secrete a range of organic acids from their mixed-acid fermentation pathways. These compounds have the potential for conversion to biofuels in downstream biological processes. As outlined in Section 4.2.2, anaerobic fungi produce H_2_ via dark fermentation. Examples of known soluble co-products of fungal H_2_ production include acetic and formic acids. The formation of these alternative end-products makes it unfeasible for the hydrogenosome metabolic pathway to achieve 100% efficiency in the conversion of carbohydrate H atoms to H_2_ gas. Previously, the maximum theoretical yield of H_2_ from dark fermentation by organisms co-producing acetic acid has been reported to be 4 mol-H_2_ mol-hexose^−1^ (Equation (1)) [133].

C_6_H_12_O_6_ + 2H_2_O → 2CH_3_COOH + 2CO_2_ + 4H_2_ (1)

**Figure 4:**
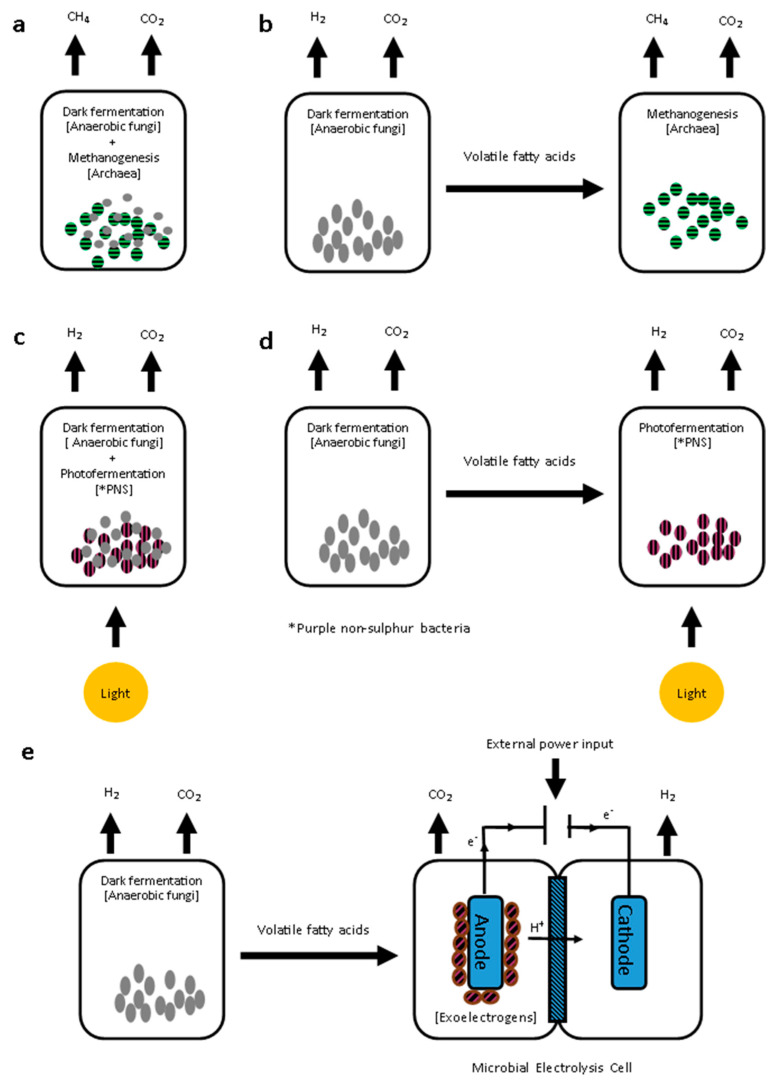
A schematic representation of the incorporation of anaerobic fungi into (**a**) single-stage biomethane production; (**b**) two-stage dark fermentation and methanogenesis; (**c**) single-stage dark fermentation and photofermentation; (**d**) two-stage dark fermentation and photofermentation; and (**e**) two-stage dark fermentation and microbial electrolysis.

There is an opportunity for industry to add value to the dark fermentation process by anaerobic fungi via integration of microorganisms which can utilise organic acids for the production of additional biofuel in the form of CH_4_ or H_2_ (Figure 4).

#### 4.3.1. Integration of Dark Fermentation with Biomethane Production

Methanogenic archaea are capable of converting acetic and formic acids into CH_4_ via Equations (2) and (3) [161].

4HCOOH → CH_4_ + 3CO_2_ + 2H_2_O (2)

CH_3_COOH → CH_4_ + CO_2_ (3)

Anaerobic fungal and methanogenesis pathways have the potential for integration as has been reported and industrialised for bacteria–archaea relationships [162]. Methanogenesis of dark fermentation products could be utilised in a single-stage bioreactor containing a mixture of anaerobic fungi and archaea (see, e.g., in [129,144]) (Figure 4a). Alternatively, a two-stage system could be used in which H_2_ is obtained from the first stage and CH_4_ collected from the subsequent second stage (Figure 4b). In the proposed two-stage system, the second stage would be physically separated and optimised for methanogenesis of compounds in the effluent from dark fermentation by anaerobic fungi. A number of conventional, continuously-fed bacterial anaerobic digestion studies have reported higher energy recovery yields of 11–37% when using two-stage systems in comparison to single-stage operation [163,164]. As previously discussed, research investigating co-culture of anaerobic fungi with methanogens strongly indicates that the presence of archaea promotes the activity of the fungus [5,6,66,138,139,141,142,143,144,145]. Comparisons of energy yields are necessary between single-stage co-culturing of anaerobic fungi and methanogens to produce solely CH_4_ or segregation of their growth in a two-stage system to obtain H_2_ and CH_4_.

#### 4.3.2. Integration of Dark Fermentation with Additional Biohydrogen Production

The soluble organic acids produced during dark fermentation are potentially suitable feed substrates for emerging downstream biological H_2_ production technologies such as microbial electrolysis cells (MEC) or photofermentation. These systems are attractive because according to stoichiometry they are theoretically capable of converting acetic acid produced from each mole of hexose consumed by the anaerobic fungus into an additional 8 mol H_2_ [165]. The combination of dark fermentation followed by either photofermentation or MEC could hypothetically achieve the complete conversion of H atoms in hexose to H_2_ fuel. This subsection discussed the concept and technological challenges of integrating dark fermentation by anaerobic fungi with MEC and photofermentation processes.

##### Photofermentation

Photofermentation is performed by purple non-sulphur (PNS) bacteria which are able to generate H_2_ by utilising dark fermentation acids in the presence of light [166]. When this group of bacteria are growing photo-heterotrophically, the production of H_2_ is catalysed by nitrogenase. PNS bacteria can oxidise organic acids (e.g., acetate) in a tricarboxylic acid cycle and shuttle the released electrons to nitrogenase via NAD/NADH and ferredoxin. The reduction of protons is energetically expensive and the ATP required to drive the nitrogenase reaction is provided by a combination of photosystem I and ATP synthase [167]. Photofermentation should be carried out in the absence of N_2_ to avoid protonation of N by nitrogenase to create ammonia at the expense of H_2_ [168]. The optimal pH and temperature for photofermentation are considered to be neutral and mesophilic, respectively [169]. Moreover, PNS bacteria are anaerobic or microaerophilic and collectively, culture conditions are similar to those required by anaerobic fungi. Therefore, similar to the suggested configurations of anaerobic fungi and methanogens, PNS bacteria could in principle perform photofermentation of fungal products in a single-stage co-culture or sequentially in a separated second-stage bioreactor (Figure 4c,d).

Integration of bacterial dark fermentation and photofermentation has previously been used to process various carbohydrates including starch [170], glucose [171], cellulose [172] and molasses [173]. It is expected that the knowledge gained from previous bacterial studies will aid the future development of photofermentation systems that include anaerobic fungi. Currently, commercialisation and wide-scale use of PNS photofermentation technology is restricted by the costs associated with high-intensity external lighting requirements and light-permeable bioreactors [169]. There is also the requirement for suitable technology to clarify dark fermentation effluents before photofermentation can occur [174]. Recent developments in the field of microalgal photobioreactors which employ the use of low-cost, low-voltage, high-intensity LED lighting, positioned in situ in the reactor show considerable promise and could be adapted for PNS bacteria and anaerobic fungal co-culture systems.

##### Microbial Electrolysis Cells (MEC)

The first successful use of a MEC to generate H_2_ was reported by Liu et al. [175] and used acetate as a carbon source. MEC are adaptions of microbial fuel cells (which typically consist of an anaerobic anode electrode chamber containing microbes and an oxygenated cathode electrode chamber). In a MEC, both electrodes are operated under anaerobic conditions and the circuit is supplemented with external power (Figure 4e). Biological oxidation of organic compounds (e.g., acetate and formate [176]) by anaerobic microbial growth at the anode releases protons and electrons. These electrons and protons differ in their routes to the cathode and travel via the electrodes or in solution, respectively. Provision of external power to the electrodes (>0.2 V [177]) creates the necessary redox potential for electrons and protons to recombine at the cathode producing H_2_ gas. Crucially, MEC systems supplied with acetate require approximately 1/10th the external power needed for abiotic H_2_ production in electrolysis of H_2_O [175]. Since the inception of MEC, advancements have been made to their design and operational parameters [178]. For example, Hou et al. [179] consistently achieved a H_2_ yield greater than 3 mol-H_2_ mol-acetate^−1^ throughout 60 days of operation while using a single chamber MEC variation in which methanogenesis was suppressed by UV irradiation. MECs have now been used to generate H_2_ from a range of bacterial dark fermentation effluents [180,181,182], paving the way for the future application of this technology to the products of dark fermentation by anaerobic fungi. Challenges faced by MEC scale-up include fouling of electrodes [183], optimisation of electrode surface area to reactor volume [184] and the prevention of methanogenesis in single-chamber reactors [185].

The discussed technologies, capable of converting organic acids from dark fermentation into additional H_2,_ are less mature than those that produce CH_4_. Nevertheless, in time, the downstream implementation of MEC or photofermentation could provide industrial opportunities that make use of the superior ability of anaerobic fungi to degrade abundantly available lignocellulose for the purpose of producing significant amounts of green H_2_.

## 5. Conclusions

Despite their biotechnological relevance and their prevalence as a critical component of ruminant biology for recovering resources from plants, anaerobic fungi remain relatively unexplored as platform organisms for lignocellulosic breakdown and biofuel production. Mimicking natural rumen and hind-gut environments in a scalable bioreactor remains a formidable challenge. The development of robust, low energy, heterologous, scalable processes that are able to make use of complex lignocellulose substrates is critical for effective process-scale production of biofuels. Except for AD reactors, most bioprocessing strategies have been well developed and optimised for aerobic microorganisms with very different growth requirements. Nevertheless, boundless opportunities arise to exploit the enzyme systems and metabolism of anaerobic fungi, whether by producing genes in heterologous platforms, by developing the means to genetically engineer the fungi directly, or by repurposing existing or developing new bioreactor designs. Such work provides an opportunity to potentially produce not only biofuels but platform molecules from one of the most abundant and renewable feedstocks on the planet.
